# Tissue Engineering and Stem Cell Therapy in Neurogenic Bladder Dysfunction: Current and Future Perspectives

**DOI:** 10.3390/medicina59081416

**Published:** 2023-08-03

**Authors:** Katarina Topoliova, Stefan Harsanyi, Lubos Danisovic, Stanislav Ziaran

**Affiliations:** 1 National Institute of Rheumatic Diseases, Nabrezie I. Krasku 4, 921 12 Piestany, Slovakia; katarina.topoliova@fmed.uniba.sk (K.T.); stefan.harsanyi@fmed.uniba.sk (S.H.); stanislav.ziaran@fmed.uniba.sk (S.Z.); 2 Department of Urology, Faculty of Medicine, Comenius University in Bratislava, Limbova 5, 833 05 Bratislava, Slovakia; 3 Institute of Medical Biology, Genetics and Clinical Genetics, Faculty of Medicine, Comenius University in Bratislava, Sasinkova 4, 811 08 Bratislava, Slovakia; 4 Regenmed Ltd., Medena 29, 811 02 Bratislava, Slovakia

**Keywords:** stem cell, tissue engineering, neurogenic bladder, bladder dysfunction

## Abstract

Tissue engineering (TE) is a rapidly evolving biomedical discipline that can play an important role in treating neurogenic bladder dysfunction and compensating for current conventional options’ shortcomings. This review aims to analyze the current status of preclinical and clinical trials and discuss what could be expected in the future based on the current state of the art. Although most preclinical studies provide promising results on the effectiveness of TE and stem cell therapies, the main limitations are mainly the very slow translation of preclinical trials to clinical trials, lack of quality research on neurogenic preconditions of neurogenic bladder dysfunction outside of the spinal cord injury and varying therapeutic methods of the existing research that lacks a standardized approach.

## 1. Introduction

The possibility of artificially creating organs has fascinated humans for a long time. Although the use of stem cells in modern medicine probably began with the treatment of myeloproliferative disorders with bone marrow transplantation in 1959, the interest has sparked significantly in the last 25 years, with the main reason being the first successful cloning of the sheep, Dolly, using a somatic cell nuclear transfer technique [1,2]. The related interest in stem cells (SC) and tissue engineering (TE) research allowed for improved and more stable access to funding. SC therapy can potentially revolutionize conventional medicine as we practice it today. TE is an interdisciplinary field that combines stem cells, scaffolds with suitable growth factors, cytokines, and chemokines to improve, replace, or regenerate organs. The self-renewal and differentiation potential of SCs can repair and regenerate damaged tissue in certain conditions.

There are four categories of SCs [3]. Embryonic SCs are undifferentiated SCs that originate from the pre-implantation blastocyst derived from 4–5-day-old human embryos. They have high proliferative capability, but since their harvesting requires human embryo destruction, their use has raised ethical concerns.

Somatic (adult) SCs are undifferentiated SCs in differentiated tissue/organs with limited capacity for differentiation. 

Mesenchymal SCs are non-blood adult SCs that can differentiate into different connective tissue cells. They also include cells from, but not limited to, adipose tissue and bone marrow.

Induced pluripotent SCs are adult SCs that are reverted to pluripotent SCs by the introduction of certain transcription factors (Oct4, Sox2, Klf4, and Myc). They turn into patient-specific SCs [4].

In recent decades, there has been relevant research aiming at SC therapy in several chronic urological conditions for which conventional therapy is unsatisfactory. 

Although the ability of SCs to repair diseased tissue has been proven in other fields, such as ophthalmology, the biological complexity of human organs was initially underestimated, and the clinical translation of SC therapy to human disease was only successful after more than 2 decades of intense research [5]. As of today, the main challenge of SC therapy and TE remains their questionable competitiveness in relation to conventional therapy when it comes to effectiveness, cost-effectiveness, and potential adverse effects.

Because the urinary tract is easily accessible via endoscopy, and due to relatively low rates of morbidity, urology remains at the top of the fields of technological innovation in medicine.

The main breakthroughs come from the application of SC therapy in animal models of bladder dysfunction, stress urinary incontinence (SUI), erectile dysfunction, and urethral injury [6,7,8,9,10,11,12]. 

As of this moment, SC therapy and TE approaches remain mostly in preclinical stages as more research and clinical trials are needed to determine the safety and efficacy of these approaches in humans.

## 2. Methods and Study Selection

**Figure 1:**
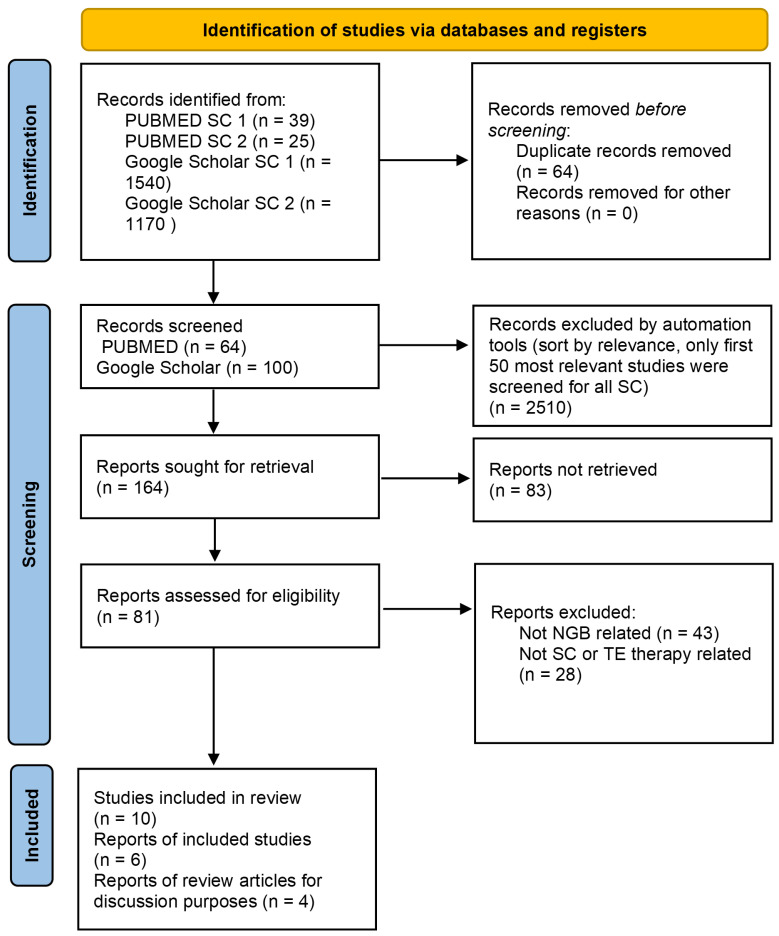
PRISMA diagram.

We followed the Preferred Reporting Items for Systematic Reviews and Meta-Analyses (PRISMA) guidelines. The search was performed (6 June 2023) in the databases PUBMED and Google Scholar. We systematically searched PUBMED databases using the following combinations of free-text words: “stem” + “cell” + “neurogenic” + “bladder” (search combination (SC) 1) and “tissue” + “engineering” + “neurogenic” + “bladder” (search combination (SC) 2). Similarly, we searched Google Scholar databases using the combination of the free-text words and the following specific terms: “stem” + “cell” + “neurogenic bladder” (SC 1), “tissue” + “engineering” + “neurogenic bladder”. All the above terms were searched with the “since 2019” filter on Google Scholar and the “publication date” filter “last 5 years” in PUBMED. After a careful selection restricted to papers published in the last 5 years in English, six preclinical studies and four review articles (for discussion purposes) met our inclusion criteria, based on their recency and circumstantiality. Identified, screened, and included articles are presented in Figure 1.

## 3. SC and TE Treatment of Neurogenic Bladder Dysfunction (NBD)

Neurogenic bladder is a collective term for disorders of the storage or excretory functions of the bladder that have neurological etiologies. These disorders can result in incontinence, urine retention, or their combination. The clinical manifestation depends on the lesion’s location and relationship to the CNS’s urinary centers. The most common causes of NBD are spinal cord injury (SCI), multiple sclerosis (MS), Parkinson’s disease (PD), and stroke.

NBD can lead to various health conditions that may greatly affect the quality of life for the patient as a source of embarrassment, discomfort, and social isolation. Aside from the inability to control urination, the NBD can lead to other severe consequences connected to urinary retention like urinary tract infections (UTIs—the inability to empty the bladder fully can cause stagnant urine to accumulate, which increases the risk of UTIs); kidney damage (if the urine backs up into the kidneys, it can cause damage to the delicate kidney tissue, leading to chronic kidney disease and even kidney failure (hydronephrosis); and bladder stones (stagnant urine in the bladder can lead to the formation of bladder stones, which can cause pain, discomfort, and urinary tract infections). Apart from issues related to urinary incontinence and retention, a neurogenic bladder may also lead to autonomic dysreflexia (a sudden and severe increase in blood pressure, which is a potentially life-threatening condition that can occur in people with spinal cord injuries) [13].

Management strategies for NBD include conservative methods (lifestyle changes, bladder retraining, and pelvic floor muscle training), pharmacological (anticholinergics and β-adrenoceptor agonists), and nonpharmacological approaches (electrical stimulation, clean intermittent catheterization, and indwelling catheters), and surgical interventions (augmentation cystoplasty) [14,15].

Despite the existing conventional therapies, improvement in voiding dysfunction has not been fully achieved and is accompanied by several side effects [16,17,18,19]. Due to the known side effects, there is a demand for alternative therapy treatments, such as SC therapy and TE. The potential of self-renewal, multilineage differentiation, site-specific migration, and tissue regeneration made SC a beneficial therapeutic tool in the treatment of several types of complications such as degenerative diseases [20]. Based on the results from previous experiments, SC transplantation is used in the management of neuro-urological diseases and is accompanied by promising outcomes [21].

As of today, there are several SC and TE approaches being investigated for bladder repair and regeneration:

- Scaffold-based approaches: These approaches involve the use of biodegradable scaffolds to support the growth and differentiation of cells [22,23,24]. Scaffold-based approaches may be further differentiated based on whether the approach uses a scaffold seeded with cells (seeded scaffold approaches) or a scaffold not seeded with cells (scaffold alone approaches). Furthermore, the seeded scaffold approaches may be generally divided into scaffolds seeded with the patient’s cells (autologous cells, such as urothelial cells and smooth muscle cells, which are implanted into the bladder to promote tissue regeneration) and scaffolds seeded with other cells (allogeneic cells, which are obtained from a donor who is genetically different from the patient but which are from the same species or xenogeneic cells obtained from a donor of a different species). Seeding a scaffold refers to the process of introducing cells onto or into the porous structure of the scaffold, with the scaffold acting as a framework or support structure for the cells to become the desired tissue type.
- Cell-based approaches: These approaches involve the use of SCs or other cell types to either generate new bladder tissue (using SCs in scaffold-based approaches as mentioned above via seeding). Alternatively, SCs may be used without a scaffold by directly transplanting them via an injection to enhance regeneration. Researchers are exploring the use of different types of stem cells, including mesenchymal stem cells, induced pluripotent stem cells (iPSCs), and bladder progenitor cells, for bladder repair and regeneration [21,25,26].
- Gene therapy approaches: These approaches involve the use of gene therapy to promote bladder regeneration. Researchers are investigating the use of growth factors and other genes that can stimulate the growth and differentiation of bladder cells [27,28].
- 3D printing approaches: These approaches involve the use of 3D printing technology to create custom-made bladder scaffolds that can support the growth and differentiation of cells [29,30].
- Neural stem cell transplantation: Neural stem cells can be transplanted into the bladder to generate new nerve cells. These cells can be obtained from the patient’s own body or from a donor. The transplanted cells can differentiate into new nerve cells and integrate into the existing neural network in the bladder, restoring bladder function [31].
- iPSC therapy: iPSCs can be differentiated into various cell types, including nerve cells. These cells can then be transplanted into the bladder to replace the damaged nerves [32].

## 4. Examining the Potential of TE and SC Treatment of Neurogenic Bladder (NGB)

One of the main challenges in the TE of bladder tissue is creating functional urothelial and smooth muscle layers. In this regard, SCs, including neural SCs and mesenchymal SCs, have shown potential for the regeneration of bladder tissues. Neural SCs can differentiate into multiple cell types, including smooth muscle cells and urothelial cells, while mesenchymal stem cells can differentiate into smooth muscle cells and provide trophic support to promote tissue repair and regeneration. Scaffold-based approaches are also being investigated for the TE of bladder tissues. These approaches involve the use of porous biomaterials as a scaffold to support the growth and differentiation of cells into functional bladder tissues. Additionally, advances in 3D printing technology have allowed for the creation of patient-specific bladder constructs, which can be tailored to the specific needs of each patient.

TE has been explored in pediatric urology as an alternative to enterocystoplasty for the management of NBD by utilizing biodegradable scaffolds, either unseeded or seeded with primary cells, in both animal models and clinical trials [33,34,35,36,37]. Synthetic materials that have been tested in experimental and clinical settings include polyvinyl sponges, Teflon, collagen matrices, Vycryl (PGA) matrices, and silicone; however, most of them failed because of mechanical, structural, functional, and biocompatibility issues. It was soon clear that non-biodegradable synthetic scaffolds used for bladder reconstruction are usually prone to mechanical failure and urinary stone formation, while biodegradable ones can lead to fibroblast deposition, scarring, graft contracture, and reduced reservoir volume over time, especially in a non-seeded configuration. Consequently, studies were then mainly focused on biodegradable scaffolds (bladder submucosa, collagen and polyglycolic acid, small intestinal submucosa, bladder acellular matrix, amniotic membrane, etc.) for urinary bladder reconstruction, eventually involving the use of seeded cells to enhance tissue regeneration. To date, several types of scaffolds and cells have been evaluated to reconstruct the urinary bladder, but various animal models and surgical repairs have also been investigated [38]. 

Although the ideal solution is yet to be found, biodegradability seems to be a crucial feature, especially in the pediatric field. Additionally, if urothelial regeneration can be more easily obtained, muscle, nerve, and vascular regeneration cannot be achieved without the pre-seeding of a scaffold, eventually combining the use of specific growth factors. Consequently, it became clear that scaffolds working well in healthy urinary tissues could not necessarily be as effective in a diseased model in which the cells that would populate the graft were generally abnormal. Therefore, the main measure of success for the scaffold should be to demonstrate not only tissue layer regeneration but also its ability to improve the capacity and compliance of the bladder. Minimizing the effects of congenital malformations of the urinary tract remains a challenge [39]. 

Despite the progress made in urological TE, several issues still have to be faced in order to improve the results in terms of muscle regeneration, the limitation of complications, and the functional restoration of the urethra and urinary bladder, especially in the case of pediatric patients [22]. Future challenges lie in resolving several factors including the mechanical properties of the graft (with the intention to mimic the structure, biomechanics, and physiology of a natural bladder), the size of the graft (the consistency of reporting graft sizes in research is lacking, and extensive cell regeneration tends to occur in the peripheral area of the graft, while the regeneration in the center is not necessarily sufficient), the vascularization of the graft (difficulties in the neovascularization of larger grafts), the fibrotic reaction of the graft (the implantation of bladder scaffolds typically triggers a fibrotic reaction), and the innervation of bioengineered bladders (although patients with neurogenic bladder undergo bladder augmentation, in order to expand urinary bladder capacity and decrease bladder pressure) [40].

## 5. Results

According to the study selection process, six articles were selected to be included in this systematic review. Most of the recent work has focused on novel sources of SCs, their improvement by engineering strategies, and improving the vehicles for their in vivo application. The reviewed studies may be divided into two main categories based on their different areas of focus. The first group of recent studies focused on bladder reconstruction using scaffold-based approaches, while the second group examined the neuro-regenerative capabilities of SCs to facilitate healing after the transplantation of SCs following SCI-induced NBD.

Considering the first group of studies, Chen, J., Wang, L., Liu, M. et al. have recently studied the implantation of adipose-derived mesenchymal stem cell (ADSC) sheets into an SCI rat model by preparing ADSC sheets from the adipose tissue of Sprague Dawley (SD) rats using temperature-responsive cell culture dishes with a result of promoting axonal regeneration and restoring bladder function after a spinal cord injury [25]. Adult female SD rats were subjected to SCI by transection at the T10 level and administered ADSC sheets or a gelatin sponge (the control group). The ADSC sheet transplantation significantly improved voiding function recovery in rats after SCI and is therefore a promising cell delivery treatment option for NGB related to SCI.

Another novel strategy was used by Song, Y., Li., Y., Tian, M. et al. who used an antibody-conjugated small intestine submucosa to capture urine-derived stem cells for bladder repair in a rabbit model [23]. The authors developed an anti-CD29 antibody-crosslinked submucosa of a small intestine scaffold (AC-SIS), capable of specifically capturing urine-derived stem cells (USCs), that possesses sound biocompatibility. The research found that the AC-SIS promoted in situ tissue regeneration by facilitating the repair of bladder epithelium, smooth muscle regeneration, and angiogenesis. This is a new design, with a new application, for endogenous-stem-cell-capturing scaffolds that provides a new strategy for bladder repair.

Regarding the second group of studies, a recently used approach for the restoration of bladder function after acute SCI studied the use of human umbilical cord mesenchymal SCs. The study was conducted by Li, J., Huang, J., Chen, L. et al. [31]. In a Sprague Dawley (SD) rat model of SCI, hUC-MSCs prepared from three fresh umbilical cord samples from healthy newborn fetuses were injected into the spinal cord of the SCI rats 8 days after SCI induction. The research found that hUC-MSCs contributed to the reconstruction of bladder function after SCI and partially restored the motor function of SCI rats by ameliorating the destructive lesions. Compared with control rats receiving a sham operation, the bladder capacity and urination volume of SCI-modeled rats were elevated, with especially higher residual urine volume and urination efficiency, whilst the transplantation of hUC-MSCs alleviated SCI-induced increased bladder capacity, residual urine output, and urinary output, thereby improving the efficiency of urination. In addition, hUC-MSCs reduced the SCI-induced gain of bladder weight. This research provided the first evidence that hUC-MSCs were likely to play pivotal roles in the reconstruction of bladder functions after acute SCI despite the limitations, including the small sample size of the study of only one animal species. The authors also did not research how the hUC-MSCs regulate the immune system at the injection site.

In a study published in 2020, Liang, CC., Shaw, SW.S., Ko, YS. et al. used human amniotic fluid as a source of SCs and studied the effect of human amniotic fluid stem cell (hAFSC) transplantation on the recovery of bladder dysfunction in SCI rats [26]. The transplantation to the transection site occurred on the 9th day after the SCI surgery. The study results demonstrated improved bladder weights of the SCI rats after the hAFSC transplantation. The dysfunctions after SCI induction, bladder elastin, and collagen level also improved after hAFSC transplantation. However, the authors pointed out that the increase in bladder weight may be due to increased bladder wall muscle and a larger urothelium. Additionally, connective tissue and fibrotic changes accumulated in the bladder walls, which adversely affected detrusor function and the bladder’s capacity to empty and might result in irreversible bladder damage even after hAFSC transplantation. A further limitation of the study was that the hAFSCs were used at a single density, while it is possible that a higher density or repeated transplantation could have better effects on SCI-induced bladder dysfunction. Also, the functional and morphological alterations in the bladders of SCI rats were examined at days 7 and 28 after transplantation, but better results could have been obtained if cystometric analysis was performed at a longer period after transplantation.

Another study published in 2020 and performed by Zhu GQ, Jeon SH, Lee KW et al. focused on improving neurogenic bladder after a pelvic nerve injury using engineered SCs by overexpressing stromal-cell-derived factor-1 (SDF-1) secreted by immortalized mesenchymal stem cells (imMSCs) on a neurogenic bladder in a rat model [41]. In this study, primary bone marrow mesenchymal stem cells (BM-MSCs) were transfected into immortalized upregulated SDF-1-engineered BM-MSCs or immortalized normal SDF-1-engineered BM-MSCs. Neurogenic bladder rats induced by bilateral pelvic nerve (PN) transection were treated with engineered imMSCs or a sham operation. The treatment improved the neurogenic bladder and evidently stimulated the recovery of the bladder wall in neurogenic bladder rats. The recovery of injured nerves was more effective in the treated group than in other groups. High SDF-1 expression improved the levels of vascular endothelial growth factor and basic fibroblast growth factor. The research demonstrated that the overexpression of SDF-1 induced additional MSC homing to the injured tissue, which improved the neurogenic bladder by accelerating the restoration of injured nerves in a rat model. Although the viability of MSCs before and after transfection was not assessed, which partially decreased the accuracy of the experiment, the experiments suggested a new therapeutic strategy using MSCs for neurogenic bladder treatment and provided a theoretical and experimental basis for future clinical treatment.

Salehi-pourmehr, H., Rahbarghazi, R., Mahmoudi J. et al. examined the outcomes of the intra-bladder wall transplantation of bone marrow mesenchymal stem cells (BM-MSCs) in a rat model and concluded that the transplantation led to improved urinary bladder dysfunction following SCI [21]. A total of 42 female Wistar rats were randomly divided into six groups (7 in each) and subjected to complete and incomplete spinal cord transection by a laminectomy. Four weeks after the SCI operation, BM-MSCs were transplanted in six areas of the bladder muscle in rats with complete SCI (cSCI) and hemi-SCI (hSCI) groups. In the rats from the sham operation, the cSCI and hSCI negative control groups, normal saline was injected instead of BM-MSCs. Four weeks following cell transplantation, rats were subjected to conscious urodynamics for voiding function assessment. The urinary dysfunction associated with SCI was improved following the direct injection of autologous BM-MSC transplantation to the bladder wall in the chronic phase of SCI injury, but a statistically significant result was only seen in the hSCI group. BM-MSCs may therefore represent another potential stem cell source for bladder reconstitution after SCI, albeit not in the complete SCI.

## 6. Discussion

While reconstructive urology needs advanced therapeutic modalities utilizing recent biotechnological advances to improve current treatment effectiveness, and while TE focusing on manipulation with cells and biomaterials ideally fits the modern reconstructive urology development pathway, it currently remains in question whether TE may be successfully applied outside the experimental field as this method is marginally used in urology. Despite decades of research, TE is still in the early stages of revolutionizing urology. Only a forward-looking approach to TE research and reliable study reports with repeatable outcomes will accelerate this process [42]. The latest reviews that systematized TE and SC treatment of NBD in preclinical stages showed a general scarcity of research related to neurological causes of NBD resulting from MS, PD, or stroke. They also showed a significant prevalence of studies performed on rat models compared to other animal models. 

A recently published review performed by Ferreira, A., Nascimento, D., and Cruz, C.D. focusing on NBD examined molecular mechanisms operating in animal models of neurogenic detrusor overactivity with a focus on which animal models are most used for research related to the treatment of NBD [21]. Based on the results of the review, the preferred animal models for NBD research purposes were rats (71%), followed by mice (23%) and rabbits (4%), and only one study was performed on non-human primates [43]. The review concluded that most studies concerning molecular mechanisms associated with this pathology are based on traumatic SCI models, and there is much less information about other diseases connected to NBD. More focus is needed on other disorders that lead to NBD to provide a better understanding of the pathophysiological mechanisms leading to NBD, which is important for the development of new therapies targeting these patients’ quality of life. 

Similarly, according to a systematic review on stem cell therapy for NBD in rodent models published in 2020, out of 20 studies eligible for review, 15 were conducted on SCI, 2 were conducted on PD, 2 were conducted on stroke, and 1 was conducted on MS [44]. The review concluded that the common SC sources used for therapeutical purposes were neural progenitor cells, bone marrow mesenchymal SCs, human amniotic fluid SCs, and human umbilical cord blood SCs. There was a significant improvement of micturition pressure in both contusion and transaction SCI models 4- and 8 weeks post-SC transplantation. Residual urine volume, micturition volume, and bladder capacity were improved 28 days after SC transplantation only in the transaction model of SCI. Non-voiding contraction recovered only 56 days following cell transplantation in the contusion model. According to the outcomes of the review, high-quality experiments to reduce the potential risk of bias are necessary for an improved understanding of bladder recovery. Also, due to limited studies on the relation of other neurological disorders such as PD, stroke, and MS with NBD, additional studies with the modified methodology to reduce the risk of bias are needed to prove the underlying mechanism and to achieve an appropriate approach for bladder recovery.

Recent reviews on the clinical application of SC therapy also did not bring significant conclusive results, mainly due to a lack of a standardized approach.

Salehi-pourmehr, H., Nouri, O., Naseri, A. et al. performed a systematic review of the clinical application of SC therapy in neurogenic bladder in 2021 [45]. The review aimed to investigate the effect of SC therapy on the management of NBD in four neurological diseases, including SCI, PD, MS, and stroke, in the clinical setting. The review concluded, that, although most clinical trials provide evidence of the safety and effectiveness of MSCs in the management of NBD, the meta-analysis results did not show a significant improvement; however, the interpretation of study results was difficult because of the lack of placebo controls.

Comparably, a review performed in 2020 by Kim SJ, Cho YS, Park JM et al. of studies related to SC therapy for neurogenic bladder after SCI concluded that SC therapy is a promising therapeutic option for neurogenic bladder related to SCI, and many preclinical and clinical studies have been conducted to demonstrate the efficacy and safety of SC therapy [46]. Several clinical trials have demonstrated improvements in bladder function. However, clear evidence is lacking because most of the extant clinical trials were not of high quality, and therapeutic methods varied among the studies. Therefore, there is a pressing need for further studies to demonstrate evidence of the therapeutic potential of stem cell therapy and to enable the translation of SC therapy to real-world practice.

Although showing promise, there are major issues in SC therapy that correlate with the cell survival, dynamic growth, and regeneration potential of transplanted cells in the long-term outcome given the existing concern about cellular rejection by the adaptive immune response in the host tissues since a large number of transplant cells die within hours and days following administration [47,48].

Taking into consideration decades of preclinical research of SC therapy and TE and almost no clinical research, the transition from preclinical research to the clinical stages seems very difficult for the scientific community. 

The reasons probably stem from the fact that the potential risks and side effects of SC therapy and TE are not yet fully known. Some potential risks have been identified based on preclinical studies and early clinical trials [49,50,51]. 

One potential risk is the development of tumors or other abnormal tissue growth, which has been observed in some preclinical studies using SC therapies, although most results of mesenchymal stem cell administration in vivo have confirmed their safety and showed promising beneficial outcomes [52]. There is also a risk of immune rejection or immune reaction to the implanted stem cells or engineered tissues, which could lead to inflammation, tissue damage, or other complications [53]. 

Another potential risk is the introduction of infections, particularly if the patient has a compromised immune system. There is also a risk of surgical complications or damage to surrounding tissues during the implantation procedure.

Finally, there is a risk of adverse reactions to the drugs or other agents used to promote stem cell differentiation or tissue growth (growth factors, cytokines, immunomodulatory drugs, etc.), including allergic reactions or toxicity.

Referring to the above-listed potential risks and side effects, it is difficult to initiate a clinical trial. The motivation in neurogenic bladder patients to seek alternative therapy options is also not comparable to patients suffering from other life-changing conditions (e.g., immobility and blindness), which might make the recruitment difficult.

The mentioned difficult transition between preclinical and clinical studies may be the reason for the current vast discrepancy between the volume of preclinical trials and their application in humans. The existing clinical trials related to neurogenic bladder and SC therapy come mostly as derivative secondary outcomes of clinical trials aimed at the treatment of other primary consequences of SCI [54,55,56,57,58,59,60,61,62]. Furthermore, it is difficult to draw definitive conclusions even from the mentioned existing clinical trials as most of the previous clinical trials were not randomized trials, did not have a control group, and included a small number of patients. Moreover, the dose, route of administration, and timing were different in each of the studies. No studies demonstrated functional recovery of voiding as the patients still had persistent urinary incontinence and a continuing need for clean intermittent catheterization. Similarly, no study showed improvements in sensory and motor function after SC therapy. Therefore, further studies are necessary to demonstrate clear evidence regarding SC therapy and the appropriate direction for the standardization of therapeutic methods [46]. Recent developments in the transition to clinical trials of non-neurogenic patients show that SC therapy may be closer to more intensive clinical trial stages for NGB patients as well [63].

Although the preclinical studies have had very encouraging results, the excitement has also led to the use of unregulated SC therapy and products due to the general lack of regulation of SC therapy and related unscientific administration of SC therapy to patients [64]. The most recent preclinical research, in the last three years, focuses mostly on rodent models of NGB following SCI, while recent targeted research following other conditions that lead to NGB (stroke, PD, and MS) is lacking. This may be explained by the easier accessibility of inducing SCI in preclinical animal models compared to other NGB preconditions.

Referring to the current state of preclinical and clinical trials related to SC and TE treatment of NBD, the main setbacks are the lack of quality research on neurogenic preconditions of NGB outside of SCI and varying therapeutic methods of the existing research that lack a standardized approach.

## 7. Conclusions

Given the high volume of SCI preclinical research and some clinical studies, the near future may provide us with breakthroughs to the clinical phase of research, using SC treatment methods previously approved for other diseases [65]. The hope is that the focus of preclinical research will provide greater detail in examining the adverse effects of SC and TE treatment, which could then accelerate the much-needed transition to clinical trials (and the quality and standardization of existing clinical trials) after decades of preclinical focus on animal models.

If the excitement to make the transition to clinical trials faster in the most researched preclinical aspects of NGB treatment via SC and TE (e.g., SCI rodent models) seems premature, there is also room to grow for the quality of preclinical stages. Despite the strict regulation and higher costs related to using primates in research, non-human primate (NHP) models may provide more concrete and high-quality results for safe SC and TE preclinical trials due to the genetic and physiological (e.g., bigger graft size for scaffold TE in bladders) similarities to humans. The difficulties of the preclinical application of SC and TE treatment in the NHP models may be overcome by answering calls for the free sharing of cell lines and protocols between research groups [66]. Definitive conclusions on the safety of SC and TE treatments are needed to elevate the confidence of the scientific community to pursue the potential benefits of stem cell research in regenerative medicine and bring the anticipated revolution in the treatment of degenerative conditions that could significantly improve the quality of life of NGB patients.

## Data Availability

Not applicable.

## References

[1] Thomas E.D., Lochte H.L., Cannon J.H., Sahler O.D., Ferrebee J.W. (1959). Supralethal whole body irradiation and isologous marrow transplantation in man. J. Clin. Invest..

[2] Campbell K.H.S., McWhir J., Ritchie W.A., Wilmut I. (1996). Sheep Cloned by Nuclear Transfer from a Cultured Cell Line. Nature.

[3] Home | STEM Cell Information. https://stemcells.nih.gov/.

[4] Thomson J.A., Itskovitz-Eldor J., Shapiro S.S., Waknitz M.A., Swiergiel J.J., Marshall V.S., Jones J.M. (1998). Embryonic Stem Cell Lines Derived from Human Blastocysts. Science.

[5] Rama P., Matuska S., Paganoni G., Spinelli A., De Luca M., Pellegrini G. (2010). Limbal Stem-Cell Therapy and Long-Term Corneal Regeneration. N. Engl. J. Med..

[6] Woo L.L., Tanaka S.T., Anumanthan G., Pope J.C., Thomas J.C., Adams M.C., Brock J.W., Bhowmick N.A. (2011). Mesenchymal Stem Cell Recruitment and Improved Bladder Function after Bladder Outlet Obstruction: Preliminary Data. J. Urol..

[7] Nishijima S., Sugaya K., Miyazato M., Kadekawa K., Oshiro Y., Uchida A., Hokama S., Ogawa Y. (2007). Restoration of Bladder Contraction by Bone Marrow Transplantation in Rats with Underactive Bladder. Biomed. Res..

[8] Levanovich P.E., Diokno A., Hasenau D.L., Lajiness M., Pruchnic R., Chancellor M.B. (2015). Intradetrusor Injection of Adult Muscle-Derived Cells for the Treatment of Underactive Bladder: Pilot Study. Int. Urol. Nephrol..

[9] Vinarov A., Atala A., Yoo J., Slusarenco R., Zhumataev M., Zhito A., Butnaru D. (2018). Cell Therapy for Stress Urinary Incontinence: Present-Day Frontiers. J. Tissue Eng. Regen. Med..

[10] Williams J.K., Badlani G., Dean A., Lankford S., Poppante K., Criswell T., Andersson K.-E. (2016). Local versus Intravenous Injections of Skeletal Muscle Precursor Cells in Nonhuman Primates with Acute or Chronic Intrinsic Urinary Sphincter Deficiency. Stem. Cell Res. Ther..

[11] Raya-Rivera A., Esquiliano D.R., Yoo J.J., Lopez-Bayghen E., Soker S., Atala A. (2011). Tissue-Engineered Autologous Urethras for Patients Who Need Reconstruction: An Observational Study. Lancet.

[12] Osman N.I., Patterson J.M., MacNeil S., Chapple C.R. (2014). Long-Term Follow-up after Tissue-Engineered Buccal Mucosa Urethroplasty. Eur. Urol..

[13] Sakaibara R., Uchiyama T., Kuwabara S., Kawaguchi N., Nemoto I., Nakata M., Hattori H. (2001). Autonomic Dysreflexia Due to Neurogenic Bladder Dysfunction; an Unusual Presentation of Spinal Cord Sarcoidosis. J. Neurol. Neurosurg Psychiatry.

[14] Sturm R.M., Cheng E.Y. (2016). The Management of the Pediatric Neurogenic Bladder. Curr. Bladder Dysfunct. Rep..

[15] Lucas E. (2019). Medical Management of Neurogenic Bladder for Children and Adults: A Review. Top. Spinal Cord Inj. Rehabil..

[16] Hajebrahimi S., Chapple C.R., Pashazadeh F., Salehi-Pourmehr H. (2019). Management of Neurogenic Bladder in Patients with Parkinson’s Disease: A Systematic Review. Neurourol Urodyn.

[17] Wyndaele J.J., Madersbacher H., Kovindha A. (2001). Conservative Treatment of the Neuropathic Bladder in Spinal Cord Injured Patients. Spinal Cord.

[18] Gajewski J.B., Schurch B., Hamid R., Averbeck M., Sakakibara R., Agrò E.F., Dickinson T., Payne C.K., Drake M.J., Haylen B.T. (2018). An International Continence Society (ICS) Report on the Terminology for Adult Neurogenic Lower Urinary Tract Dysfunction (ANLUTD). Neurourol Urodyn.

[19] de Sèze M., Ruffion A., Denys P., Joseph P.-A., Perrouin-Verbe B. (2007). GENULF The Neurogenic Bladder in Multiple Sclerosis: Review of the Literature and Proposal of Management Guidelines. Mult. Scler..

[20] Azizi R., Aghebati-Maleki L., Nouri M., Marofi F., Negargar S., Yousefi M. (2018). Stem Cell Therapy in Asherman Syndrome and Thin Endometrium: Stem Cell- Based Therapy. Biomed. Pharmacother..

[21] Salehi-Pourmehr H., Rahbarghazi R., Mahmoudi J., Roshangar L., Chapple C.R., Hajebrahimi S., Abolhasanpour N., Azghani M.-R. (2019). Intra-Bladder Wall Transplantation of Bone Marrow Mesenchymal Stem Cells Improved Urinary Bladder Dysfunction Following Spinal Cord Injury. Life Sci..

[22] Wang X., Zhang F., Liao L. (2021). Current Applications and Future Directions of Bioengineering Approaches for Bladder Augmentation and Reconstruction. Front. Surg..

[23] Song Y.-T., Li Y.-Q., Tian M.-X., Hu J.-G., Zhang X.-R., Liu P.-C., Zhang X.-Z., Zhang Q.-Y., Zhou L., Zhao L.-M. (2022). Application of Antibody-Conjugated Small Intestine Submucosa to Capture Urine-Derived Stem Cells for Bladder Repair in a Rabbit Model. Bioact. Mater..

[24] Song Y.-T., Dong L., Hu J.-G., Liu P.-C., Jiang Y.-L., Zhou L., Wang M., Tan J., Li Y.-X., Zhang Q.-Y. (2023). Application of Genipin-Crosslinked Small Intestine Submucosa and Urine-Derived Stem Cells for the Prevention of Intrauterine Adhesion in a Rat Model. Compos. Part B Eng..

[25] Chen J., Wang L., Liu M., Gao G., Zhao W., Fu Q., Wang Y. (2022). Implantation of Adipose-Derived Mesenchymal Stem Cell Sheets Promotes Axonal Regeneration and Restores Bladder Function after Spinal Cord Injury. Stem. Cell Res. Ther..

[26] Liang C.-C., Shaw S.-W.S., Ko Y.-S., Huang Y.-H., Lee T.-H. (2020). Effect of Amniotic Fluid Stem Cell Transplantation on the Recovery of Bladder Dysfunction in Spinal Cord-Injured Rats. Sci. Rep..

[27] Sievert K.-D., Renninger M., Füllhase C., Liao L., Madersbacher H. (2019). Other New Developments: Use of Stem Cells and Gene Therapy. Neurourology: Theory and Practice.

[28] Liao L. (2015). Evaluation and Management of Neurogenic Bladder: What Is New in China?. Int. J. Mol. Sci..

[29] Zhao Y., Liu Y., Dai Y., Yang L., Chen G. (2022). Application of 3D Bioprinting in Urology. Micromachines.

[30] Chowdhury S.R., Keshavan N., Basu B. (2021). Urinary Bladder and Urethral Tissue Engineering, and 3D Bioprinting Approaches for Urological Reconstruction. J. Mater. Res..

[31] Li J., Huang J., Chen L., Ren W., Cai W. (2022). Human Umbilical Cord Mesenchymal Stem Cells Contribute to the Reconstruction of Bladder Function after Acute Spinal Cord Injury via P38 Mitogen-Activated Protein Kinase/Nuclear Factor-Kappa B Pathway. Bioengineered.

[32] Kibschull M., Nguyen T.T.N., Chow T., Alarab M., Lye S.J., Rogers I., Shynlova O. (2023). Differentiation of Patient-Specific Void Urine-Derived Human Induced Pluripotent Stem Cells to Fibroblasts and Skeletal Muscle Myocytes. Sci. Rep..

[33] Kikuno N., Kawamoto K., Hirata H., Vejdani K., Kawakami K., Fandel T., Nunes L., Urakami S., Shiina H., Igawa M. (2009). Nerve Growth Factor Combined with Vascular Endothelial Growth Factor Enhances Regeneration of Bladder Acellular Matrix Graft in Spinal Cord Injury-Induced Neurogenic Rat Bladder. BJU Int..

[34] Obara T., Matsuura S., Narita S., Satoh S., Tsuchiya N., Habuchi T. (2006). Bladder Acellular Matrix Grafting Regenerates Urinary Bladder in the Spinal Cord Injury Rat. Urology.

[35] Urakami S., Shiina H., Enokida H., Kawamoto K., Kikuno N., Fandel T., Vejdani K., Nunes L., Igawa M., Tanagho E.A. (2007). Functional Improvement in Spinal Cord Injury-Induced Neurogenic Bladder by Bladder Augmentation Using Bladder Acellular Matrix Graft in the Rat. World J. Urol..

[36] Atala A., Bauer S.B., Soker S., Yoo J.J., Retik A.B. (2006). Tissue-Engineered Autologous Bladders for Patients Needing Cystoplasty. Lancet.

[37] Joseph D.B., Borer J.G., De Filippo R.E., Hodges S.J., McLorie G.A. (2014). Autologous Cell Seeded Biodegradable Scaffold for Augmentation Cystoplasty: Phase II Study in Children and Adolescents with Spina Bifida. J. Urol..

[38] Casarin M., Morlacco A., Dal Moro F. (2022). Tissue Engineering and Regenerative Medicine in Pediatric Urology: Urethral and Urinary Bladder Reconstruction. Int. J. Mol. Sci..

[39] Khan K., Ahram D.F., Liu Y.P., Westland R., Sampogna R.V., Katsanis N., Davis E.E., Sanna-Cherchi S. (2022). Multidisciplinary Approaches for Elucidating Genetics and Molecular Pathogenesis of Urinary Tract Malformations. Kidney Int..

[40] Serrano-Aroca Á., Vera-Donoso C.D., Moreno-Manzano V. (2018). Bioengineering Approaches for Bladder Regeneration. Int. J. Mol. Sci..

[41] Zhu G.Q., Jeon S.H., Lee K.W., Cho H.J., Ha U.-S., Hong S.-H., Lee J.Y., Kwon E.B., Kim H.-J., Lee S.M. (2020). Engineered Stem Cells Improve Neurogenic Bladder by Overexpressing SDF-1 in a Pelvic Nerve Injury Rat Model. Cell Transpl..

[42] Ławkowska K., Rosenbaum C., Petrasz P., Kluth L., Koper K., Drewa T., Pokrywczynska M., Adamowicz J. (2022). Trauma and Reconstructive Urology Working Party of the European Association of Urology Young Academic Urologists Tissue Engineering in Reconstructive Urology-The Current Status and Critical Insights to Set Future Directions-Critical Review. Front. Bioeng. Biotechnol..

[43] Pritchard S., Jackson M.J., Hikima A., Lione L., Benham C.D., Chaudhuri K.R., Rose S., Jenner P., Iravani M.M. (2017). Altered Detrusor Contractility in MPTP-Treated Common Marmosets with Bladder Hyperreflexia. PLoS ONE.

[44] Salehi-Pourmehr H., Hajebrahimi S., Rahbarghazi R., Pashazadeh F., Mahmoudi J., Maasoumi N., Sadigh-Eteghad S. (2020). Stem Cell Therapy for Neurogenic Bladder Dysfunction in Rodent Models: A Systematic Review. Int. Neurourol. J..

[45] Salehi-Pourmehr H., Nouri O., Naseri A., Roshangar L., Rahbarghazi R., Sadigh-Eteghad S., Mahmoudi J., Mostafaei H., Roshandel M.R., Hoseini L. (2022). Clinical Application of Stem Cell Therapy in Neurogenic Bladder: A Systematic Review and Meta-Analysis. Int. Urogynecol. J..

[46] Kim S.J., Cho Y.S., Park J.M., Na Y.G., Kim K.H. (2020). Stem Cell Therapy for Neurogenic Bladder After Spinal Cord Injury: Clinically Possible?. Int. Neurourol. J..

[47] Negro A., Hilaire C.S., Boehm M., Hayat M.A. (2012). Cell-Based Regenerative Therapies: Role of Major Histocompatibility Complex-1 Antigen. Stem Cells and Cancer Stem Cells, Volume 3: Stem Cells and Cancer Stem Cells, Therapeutic Applications in Disease and Injury: Volume 3.

[48] Robey T.E., Saiget M.K., Reinecke H., Murry C.E. (2008). Systems Approaches to Preventing Transplanted Cell Death in Cardiac Repair. J. Mol. Cell Cardiol..

[49] Baranovskii D.S., Klabukov I.D., Arguchinskaya N.V., Yakimova A.O., Kisel A.A., Yatsenko E.M., Ivanov S.A., Shegay P.V., Kaprin A.D. (2022). Adverse Events, Side Effects and Complications in Mesenchymal Stromal Cell-Based Therapies. Stem. Cell Investig..

[50] Herberts C.A., Kwa M.S.G., Hermsen H.P.H. (2011). Risk Factors in the Development of Stem Cell Therapy. J. Transl. Med..

[51] Harris A.R., Walker M.J., Gilbert F. (2022). Ethical and Regulatory Issues of Stem Cell-Derived 3-Dimensional Organoid and Tissue Therapy for Personalised Regenerative Medicine. BMC Med..

[52] Lukomska B., Stanaszek L., Zuba-Surma E., Legosz P., Sarzynska S., Drela K. (2019). Challenges and Controversies in Human Mesenchymal Stem Cell Therapy. Stem. Cells Int..

[53] Djouad F., Plence P., Bony C., Tropel P., Apparailly F., Sany J., Noël D., Jorgensen C. (2003). Immunosuppressive Effect of Mesenchymal Stem Cells Favors Tumor Growth in Allogeneic Animals. Blood.

[54] Neuralstem Inc. (2017). A Phase 1, Open-Label, Single-Site, Safety Study of Human Spinal Cord-Derived Neural Stem Cell Transplantation for the Treatment of Chronic SCI. https://clinicaltrials.gov/ct2/show/NCT01772810.

[55] (2020). Stem Cells Arabia Transplantation of Purified Autologous Bone Marrow- or Leukapheresis-Derived CD34+ and CD133+ for Patients With Spinal Cord Injuries: A Long-Term Comparative Evaluation of Safety and Efficacy Study. https://clinicaltrials.gov/ct2/show/NCT02687672.

[56] (2020). Banc de Sang i Teixits A Phase I/IIa, Randomized, Double-Blind, Single-Dose, Placebo Controlled, Two-Way Crossover Clinical Trial to Assess the Safety and to Obtain Efficacy Data in Intrathecal Administration of Expanded Wharton’s Jelly Mesenchymal Stem Cells in Chronic Traumatic Spinal Cord Injury. https://clinicaltrials.gov/ct2/show/NCT03003364.

[57] Rong L. (2019). The Effect of Intrathecal Transplantation of Umbilical Cord Mesenchymal Stem Cells in Patients With Early Stage of Chronic Spinal Cord Injury: A Multicenter, Randomized, Controlled Trial. https://clinicaltrials.gov/ct2/show/NCT03521323.

[58] Rong L. (2019). The Effect of Intrathecal Transplantation of Umbilical Cord Mesenchymal Stem Cells in Patients With Sub-Acute Spinal Cord Injury: A Multicenter, Randomized, Controlled Trial. https://clinicaltrials.gov/ct2/show/NCT03521336.

[59] (2011). General Hospital of Chinese Armed Police Forces Efficacy Difference Between Rehabilitation Therapy and Umbilical Cord Derived Mesenchymal Stem Cells Transplantation in Patients With Acute or Chronic Spinal Cord Injury in China. https://clinicaltrials.gov/ct2/show/NCT01393977.

[60] Jesús Vaquero Crespo M.D. (2018). Intrathecal Administration (Pattern 100/3) of Expanded Autologous Adult Bone Marrow Mesenchymal Stem Cells in Established Chronic Spinal Cord Injuries. https://clinicaltrials.gov/ct2/show/NCT02570932.

[61] Jesús Vaquero Crespo M.D. (2019). Phase I Pilot Study to Evaluate the Security of Local Administration of Autologous Stem Cells Obtained From the Bone Marrow Stroma, in Traumatic Injuries of the Spinal Cord. https://clinicaltrials.gov/ct2/show/NCT01909154.

[62] Santos R.R. (2017). dos Phase 1 Study of Autologous Bone Marrow Stem Cell Transplantation in Patients With Spinal Cord Injury. https://clinicaltrials.gov/ct2/show/NCT01325103.

[63] Gilleran J., Diokno A.C., Ward E., Sirls L., Hasenau D., Giordano J., Shea E., Bartolone S.N., Lamb L.E., Chancellor M.B. (2021). Improved Global Response Outcome after Intradetrusor Injection of Adult Muscle-Derived Cells for the Treatment of Underactive Bladder. Int. Urol. Nephrol..

[64] Berkowitz A.L., Miller M.B., Mir S.A., Cagney D., Chavakula V., Guleria I., Aizer A., Ligon K.L., Chi J.H. (2016). Glioproliferative Lesion of the Spinal Cord as a Complication of “Stem-Cell Tourism”. N. Engl. J. Med..

[65] Xu P., Yang X. (2019). The Efficacy and Safety of Mesenchymal Stem Cell Transplantation for Spinal Cord Injury Patients: A Meta-Analysis and Systematic Review. Cell Transpl..

[66] Rodriguez-Polo I., Behr R. (2022). Non-Human Primate Pluripotent Stem Cells for the Preclinical Testing of Regenerative Therapies. Neural. Regen. Res..

